# O.4.1-2 Linking lymphocyte T phenotype to physical capacities in a geriatric population: the VIF-APAS study

**DOI:** 10.1093/eurpub/ckad133.167

**Published:** 2023-09-11

**Authors:** Anne-Sophie Rousseau, Isabelle Mothe-Satney, Frederic Chorin, Agnès Loubat, Milly Lovera, Julien Mercier, Christian Roux, Raphael Zory, Emeline Michel

**Affiliations:** Université Cote d'Azur, France; CNRS UMR 7277/INSERM UMR 1091, iBV, France; LAHMESS UPR 6312, France; Université Cote d'Azur, France; CNRS UMR 7277/INSERM UMR 1091, iBV, France; LAHMESS UPR 6312, France; Centre Hospitalier Universitaire de Nice; anne-sophie.rousseau@univ-cotedazur.fr; Université Cote d'Azur, France; CNRS UMR 7277/INSERM UMR 1091, iBV, France; Université Cote d'Azur, France; Centre Hospitalier Universitaire de Nice; anne-sophie.rousseau@univ-cotedazur.fr; Université Cote d'Azur, France; CNRS UMR 7277/INSERM UMR 1091, iBV, France; CNRS UMR 7277/INSERM UMR 1091, iBV, France; LAHMESS UPR 6312, France; Centre Hospitalier Universitaire de Nice; anne-sophie.rousseau@univ-cotedazur.fr; Université Cote d'Azur, France; LAHMESS UPR 6312, France; LAHMESS UPR 6312, France; Centre Hospitalier Universitaire de Nice; anne-sophie.rousseau@univ-cotedazur.fr

## Abstract

**Purpose:**

Potential mediators contributing to chronic inflammation in aging have both local and systemic impacts that likely promote physical decline. Supporting data in aged mice link modification of T cells to exercise performance leading to novel hypotheses involving the anti-inflammatory regulatory T cells (Tregs) in age-related changes in tissues. We investigated in a geriatric cohort whether physical markers of early signs of frailty and their reversibility would alter circulating Tregs prevalence and subtypes regarding their markers of senescence, function, and migration to tissues.

**Methods:**

Geriatric patients able to walk without technical help, were included in a 12-weeks adapted physical activity (APA) program (NCT04080063). Multidisciplinary tools were used to assess the various aspects of human movement and frailty (isokinetic testing, bioelectric impedancemetry, nutrition, etc.). Relative NK, CD8+ and CD4+ T cells proportions were determined using flow cytometry in extracted peripheral blood monocytes. The transcription factor (FoxP3), the IL-2 receptor (CD25), the senescence (KLRG1) and migration (CCR4) markers were detected using intracellular staining and flow cytometry to discriminate the associated cell subsets and Tregs.

**Results:**

66 women (82.8 ± 6 yrs) and 23 men (82.3 ± 4 yrs) took part in the study. Only 42% had a compliance rate in the APA program over 70%. 44%, were frail, 44% pre-frail and 12% non-frail (Fried criteria). In a representative sample (n = 19), the prevalence of NK cells was 3 times higher in frail subjects. The consideration of markers (Foxp3, CD25, and CCR4) was shown to discriminate an association with some specific physical parameters that are characteristic of neuromuscular and metabolic functions: %CCR4+ cells among NK cells was negatively related to leg extensors maximum strength, and the absence of CD25 in Tregs (CD4+Foxp3+) cells was negatively related to walking distance and speed.

**Conclusions:**

Our preliminary results that need to be confirmed seem to show differences in immune cell markers depending on the frailty status. This study will participate in determining immunometabolic biomarkers of frailty and vitality in a geriatric population. The aim is to promote personalized strategies to predict and counteract inflammaging in an early stage, and to accompany and evaluate adapted physical activity strategies.

